# Can Gratitude Help to Craft Your Career? The Role of Prosocial Motivation and Intrinsic Motivation

**DOI:** 10.3390/bs14100877

**Published:** 2024-09-30

**Authors:** Qing Zhang, Haibo Yu, Rui Xiong, Xiaolin Ge, Lei Gao

**Affiliations:** 1 School of Economics and Management, Harbin Normal University, Harbin 150025, China; zhangqingat301@mail.bnu.edu.cn; 2 School of Government, Beijing Normal University, Beijing 100875, China; yuhb@bnu.edu.cn (H.Y.); gaoleihr@mail.bnu.edu.cn (L.G.); 3 College of Educational Administration, Faculty of Education, Beijing Normal University, Beijing 100875, China; ruixiong@mail.bnu.edu.cn; 4 Business School, Curtin University, Perth 6102, Australia

**Keywords:** gratitude, prosocial motivation, intrinsic motivation, career crafting, the model of proactive motivation

## Abstract

Career crafting offers a new approach for individuals to cope with changing career situations. However, few studies have focused on personality-related antecedents that stably predict career proactivity. Additionally, as individuals’ careers are embedded in various social relationships, career crafting involves significant social interaction. Our study focuses on gratitude, which is related to social interaction, suggesting that gratitude fosters an appreciation for interpersonal relationships, providing a beneficial impetus for career crafting. A quantitative approach was adopted with a three-wave survey with 825 responses, and SPSS 26 and Mplus 8.3 were used as the analytical software for confirmatory factor analysis, hierarchical regression analysis, and path analysis. We conducted an empirical examination employing a moderated mediation model grounded in the framework of proactive motivation. The results revealed a positive correlation between gratitude and career crafting, with prosocial motivation mediating this relationship. Simultaneously, intrinsic motivation moderated the relationship between prosocial motivation and career crafting. Our study emphasizes gratitude’s role in career crafting and explores how it, along with prosocial motivations, drives proactive behaviors, responding to calls for relational work redesign and examining interactions between intrinsic and prosocial motivations in careers. It has important practical implications for individuals, organizations, and career counselors.

## 1. Introduction

In a professional environment full of opportunities and challenges today, an individual’s career is a journey characterized by ongoing and iterative processes of development and evolution [1,2]. Facing the challenges of new technologies, rapid market changes, and profound changes in society [3], an ideal career path does not exist, and individual career paths are bound to have certain changes in people’s cognition and behavior at specific life stages. We, therefore, argue that focusing on individuals as personal agencies that continuously acquire new knowledge and skills throughout the life cycle contributes to the emergence of more complex career trajectories. We choose a broader perspective [1] in this article and introduce the concept of career crafting, which encompasses positive career reflection and proactive career construction. It allows individuals to proactively adapt to these changes and pursue a more meaningful, fulfilling, and satisfying person–career fit [2,4]. However, why are some individuals more inclined to actively craft their careers in this process, while others tend to hesitate and fall into career dilemmas? These questions drive us to conduct in-depth research on personal characteristics (e.g., experience, age, personality, motivation [5]) to understand the mechanisms of career crafting.

As a proactive behavior [6,7], career crafting is affected by many factors, including contextual factors (e.g., learning the value of the job [7]; economic situation [2]; and family characteristics [8]) and individual factors (e.g., self-goal setting [9]; career demands [10]). However, personality traits, as key influencing factors of proactive career behaviors, have rarely appeared in studies on career crafting [9]. Individual traits are inherent and relatively stable qualities that come into play in the face of different situations and challenges [11]. These traits not only influence the degree to which individuals engage with others and access social support but also profoundly affect their attitudes and behaviors toward their careers [12]. Among many personality traits, why did our study choose gratitude as the focus? Gratitude has unique social and emotional connections and is correlated with favorable interpersonal relationships, collaboration with others, and social support [13]. Crafting may include interactions with social relationships in careers [14], and careers are embedded in families, organizations, and society [8]. In other words, trait gratitude may assume a significant role within the framework of career crafting. Grateful individuals tend to perceive, appreciate, and respond to the support of others, and this emotion can influence their social interactions and behaviors [15]. Grateful people tend to be more willing to help others, build trusting relationships, and actively participate in social activities [16]. Simultaneously, grateful individuals would also exhibit different preferences in career decision-making compared to the general population [17]. Therefore, investigating the impact mechanism of trait gratitude on individual career crafting is of significant importance. We presume that trait gratitude is likely to exert a profound influence on career crafting, motivating individuals to pursue meaningful careers more actively.

Gratitude is often associated with a desire for positive social interaction and cooperation [13], which is seen as a distal antecedent [18]. Thus, grateful individuals are more inclined to exhibit strong prosocial motivations and desire to collaborate with others and establish meaningful social connections. Based on the model of proactive motivation, prosocial motivation can be defined as “reason to proactivity” stimulating proactive behavior [19]. Prosocial motivation, as a motivational state [14], encourages individuals to actively participate in social interactions and seek to establish close social connections [20]. It is particularly important in careers, where career advancement often relies on effective social networks and partnerships [21,22]. Prosocial motivation can prompt individuals to actively participate in professional social activities, seek opportunities to cooperate with others [23], and encourage them to actively pursue career crafting. By selecting prosocial motivation as the mediating variable, we can more clearly understand how trait gratitude affects career crafting by stimulating prosocial motivation. Furthermore, according to the model of proactive motivation [24], we should focus not only on the “reason to” for proactivity (e.g., prosocial motivation) but also on its distal antecedents (e.g., trait gratitude). Therefore, this framework is well suited for exploring the antecedent mechanisms of career crafting.

In addition, career development represents a complex process intricately entwined with the impetus of individual intrinsic motivations. The adjustment of prosocial motivation demonstrates nuanced variations contingent upon the extent of intrinsic motivation [25]. Previous studies demonstrated that the interplay between prosocial motivation and intrinsic motivation serves as a guiding force for work-related behaviors [26]. In instances of high levels of intrinsic motivation, employees are naturally inclined or attracted to engage in their job responsibilities [20]. The decision to invest effort is grounded in personal enjoyment, thus rendering it voluntary, self-determined, and autonomous [27]. When intrinsic motivation is high, prosocial motivation motivates employees to believe that completing tasks is beneficial to their own chosen goals because they enjoy the work process [28], and it is easier to be motivated to actively pursue career changes [29]. Therefore, we regard intrinsic motivation as a key moderator, asserting that the dynamic interplay between intrinsic motivation and prosocial motivation will alter how prosocial motivation affects career crafting.

**Figure 1:**
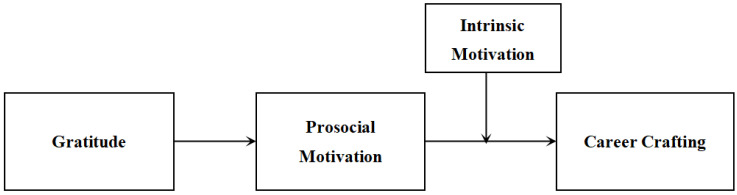
Theoretical framework. Check more details in Appendix A.

In summary, after a theoretical review and derivation, a three-wave survey collecting 825 data points was used to explore whether gratitude can help craft careers through prosocial motivation, and the results are analyzed and discussed. Exploring this path can facilitate a more profound comprehension of the favorable effects of gratitude and elucidate why individuals characterized by gratitude are more inclined to positively craft their careers. Our research will provide new insights into the fields of career development about how individual traits and motivations influence career proactive behavior. We selected a sample of basic education teachers in China for our study, as teachers may particularly benefit from carefully designing their career paths due to limited formal career development [30]. Meanwhile, due to their fundamental role in nurturing and educating, teachers are better positioned to serve both students and society when they are tasked with the ongoing responsibility of proactively updating their knowledge and refining their skills throughout their entire careers [7]. Our theoretical framework is illustrated in Figure 1.

We made several theoretical contributions. First, we discover gratitude as an individual antecedent of career crafting from individual characteristics, extending the literature on career crafting. This not only validates the applicability of the theoretical framework proposed by Parker et al. [24] but also extends its application to the career domain [18], highlighting the interconnectedness between personal traits (i.e., gratitude) and proactive motivation. Second, responding to the call of Parker et al. [14] for relational work redesign and structural support, we examine prosocial motivations and gratitude can be an important mechanism for proactive behavior. This reflects the “reason to” motivation within the proactive motivation model, demonstrating how prosocial motivation can drive proactive career behaviors. Third, our study extends the investigation of the interaction between intrinsic motivation and prosocial motivation into the career domain. This interaction supports the model of proactive motivation by showing how different motivational states can work together to enhance individual career proactivity.

## 2. Literature Review and Hypotheses

The model of proactive motivation, developed by Parker, Bindl, and Strauss [24], provides a comprehensive framework for understanding how and why individuals engage in proactive behavior in the workplace. This model integrates various motivational theories and identifies three key motivational states that drive proactive behavior: “can do” motivation, “reason to” motivation, and “energized to” motivation. Specifically, “Can do” motivation involves an individual’s belief in their capability to perform proactive tasks, emphasizing self-efficacy, control, and perceived competence. “Energized to” motivation refers to the emotional and affective states that fuel proactive behavior, with positive affect and intrinsic motivation providing the necessary energy and enthusiasm. “Reason to” motivation includes the reasons or goals driving proactive behavior, stressing the importance of clear, personal goals aligned with proactive actions. Parker et al. [24] noted that even if individuals have the ability and energy to be proactive, they still need a compelling reason to do so. Therefore, prosocial motivation, which refers to the desire to benefit others and contribute to the collective good, is considered a particularly relevant “reason to” motivation preceding proactive behavior [19].

Parker et al. [24] also emphasize that proactivity is a conscious, motivated, and goal-driven process. They suggest that more distal variables, such as individual differences (e.g., personality, knowledge, ability), contextual variations (e.g., leadership, work design, interpersonal climate), and their interactions influence proactive action by triggering motivational states that are more proximal to goals and actions. Although Parker et al. [14] highlighted the importance of structural support as a work design characteristic in the study of employee proactive behaviors, existing research on career proactivity often overlooks this aspect. Klehe et al. [18] addressed this gap by integrating the model of proactive motivation and emphasizing the role of structural support (e.g., social relationships) in fostering employee proactive behaviors.

Given the interconnectedness of an individual’s career with family, organizational, and societal relationships, our study explores antecedents of career crafting related to social-oriented traits and motivations. Gratitude, closely tied to social support [13], is chosen for its potential to inspire reliance on social networks during career changes. In the model of proactive motivation, trait gratitude serves as a distal antecedent, with prosocial motivation representing the “reason to” proactivity. It guides individuals toward cooperation, seeking support, and actions benefiting society, providing valuable psychological insights into how traits like gratitude influence career crafting.

Moreover, career crafting is delineated as a sequence of proactive behaviors undertaken by individuals to actively pursue person–career fit [2,4]. While situational factors such as career shocks, leadership, labor markets [1], and economic situations [2] undoubtedly affect career crafting, existing research largely ignores the antecedents related to individual factors [9]. Personality traits, being stable and inherent, shape decisions and behaviors, aiding individuals in adapting to career changes [11]. This provides deeper insights into why certain traits drive career crafting. Thus, building on this framework, our study focuses on gratitude as a distal factor that influences prosocial motivation, which in turn affects career crafting.

### 2.1. Gratitude, Prosocial Motivation, and Career Crafting

The concept of gratitude encompasses two distinct categories in the organizational domain: state gratitude and trait gratitude [31]. Trait gratitude, as a stable disposition, involves the consistent recognition and expression of appreciation in response to the benevolent contributions of others, particularly in facilitating positive experiences and favorable outcomes [32]. Our primary focus is on teachers’ trait gratitude, known for its enduring impact on personal well-being [33].

Gratitude contributes to the development of a psychological state focused on the well-being of others [31,34]. Concurrently, prosocial motivation is a fundamental mechanism in moral conduct linked to altruism and prosocial behavior [16]. Grateful individuals, characterized by empathy and a willingness to reciprocate [13], demonstrate altruistic tendencies, forming the foundation for the cultivation of prosocial motivation. Additionally, grateful individuals engage in social interactions to maintain positive emotions, fostering altruistic behavior and prosocial motivation [35,36]. They actively establish emotional connections with others to sustain relationships. From an evolutionary psychology standpoint, trait gratitude is seen as a tendency to form connections to navigate environmental challenges [37]. Extensive research supports the positive impact of trait gratitude on prosocial motivation [13,38]. Hence, we posit the following hypothesis:

**Hypothesis 1:**  *Gratitude positively affects prosocial motivation.*

Prosocial motivation, defined as the inclination to benefit others [20], is frequently correlated with proactive and improvement-oriented behaviors [39]. Prosocial motivation provides employees with a powerful incentive to perform physically at work. Although career crafting is an individual proactive behavior, an individual’s career is embedded in the social context and interpersonal structure [8]. Prosocial motivation drives individuals to actively participate in social interactions and build social support networks. Research shows that having a solid social support network provides individuals with emotional support, information, and resources [40,41], which can facilitate individuals to better explore and construct their careers. Thus, prosocial motivation may be a powerful factor that sustains proactivity [42].

On the other hand, prosocial motivation makes individuals pay more attention to the needs and emotions of others, fostering a deeper comprehension of their interpersonal relationships [43]. Individuals driven by prosocial motivation tend to commit themselves not only to their professional responsibilities but also to the well-being of others [44]. As they attend social interactions and perceive their value in the social network, they also construct their social network. At the same time, prosocial motivation exhibits a positive correlation with the prosocial orientation dimension of calling that sticks to one’s professional pursuits in an ever-changing career [45]. Consequently, individuals propelled by prosocial motivation are more inclined to possess a strong motivation for calling, looking for a person–career fit, and actively constructing their behavior in the process of experiencing the meaning of work [29]. Hence, we propose the following hypothesis:

**Hypothesis 2:**  *Prosocial motivation positively affects career crafting.*

Thus far, our argument posits a positive association between gratitude and prosocial motivation, further establishing a positive relationship between prosocial motivation and the endeavor of career crafting. The model of proactive motivation demonstrates that factors about “reason to” can lead to proactivity [18,24]. Specifically, gratitude can be seen as a distal factor, and it will relate to prosocial motivation as a motivational factor of “reason to” and thus make career crafting happen. Overall, in line with the model of proactive motivation, we propose that gratitude stimulates prosocial motivation, which, in turn, advances career crafting.

**Hypothesis 3:**  *Gratitude has a positive and indirect effect on career crafting via prosocial motivation.*

### 2.2. Intrinsic Motivation as a Moderator

Intrinsic motivation, fueled by interest and pleasure, drives individuals toward activities for their inherent enjoyment [46]. Previous studies have explored the synergistic effects of intrinsic motivation on the relationship between prosocial motivation and innovation [47]. Career proactive behaviors can be categorized as goal-directed actions that depend on various objectives, including self-oriented, prosocial, and pro-organizational pursuits [2]. We aim to further investigate the mechanisms through which these two motivations contribute to career proactive behavior.

Intrinsic motivation arises from one’s interest in goals and personal volition, and it is also a natural outcome of satisfying basic psychological needs [48]. Different from intrinsic motivations highlighting one’s own opinions regarding the goals, prosocial motivation views situations from others’ perspectives to get a more nuanced understanding [16,26]. Thus, prosocial motivation can be regarded as prosocial and pro-organizational pursuits for career proactive behaviors, while intrinsic motivation corresponds to self-oriented pursuits [2], suggesting that intrinsic motivation may enhance the positive link between prosocial motivation and career crafting. Specifically, activating prosocial motivation requires individuals to invest greater resources and focus on work-related interpersonal relationships and tasks [49]. Individuals with high intrinsic motivation, fueled by their genuine interest and passion for their work, are likely to produce additional resources that support proactive career activities [50]. Additionally, the generation of intrinsic motivation, associated with the fulfillment of basic psychological needs, experiences less stress when helping others [51] and can foster the positive emotions generated by prosocial motivation [52]. This dynamic process aids reflection on past career planning, envisioning future trajectories, and personal development. Conversely, a lack of intrinsic motivation hampers sustaining proactive endeavors, posing internal barriers to workforce engagement [53]. Thus, when individuals engage in career crafting with prosocial motivation, intrinsic motivation synergistically enhances this dynamic process. To sum up, we propose the following:

**Hypothesis 4:**  *Intrinsic motivation moderates the relationship between prosocial motivation and career crafting. Specifically, when intrinsic motivation is higher, the correlation between prosocial motivation and career crafting becomes stronger.*

### 2.3. The Integrated Model

Collectively, these points suggest that prosocial motivation plays a mediating role in connecting gratitude with career crafting. Our proposition infers that the association might be influenced by intrinsic motivation. We anticipate that heightened levels of intrinsic motivation would intensify the link between prosocial motivation and career crafting, thereby fostering the whole process. Accordingly, we formulate the following:

**Hypothesis 5:**  *Intrinsic motivation moderates the indirect impact of gratitude on career crafting. Specifically, a high level of intrinsic motivation can enhance the promotion influence of gratitude on career crafting via prosocial motivation.*

## 3. Method

### 3.1. Participants and Procedure

A three-wave survey was conducted using the Huajue Platform (www.huajuetech.com, accessed on 28 April 2023), allowing for a significant time gap to capture underlying factors and outcomes. This approach also mitigated common method bias concerns [54]. Teachers, with their frequent interactions with students, were chosen as suitable samples for this study [55]. The self-reported responses were gathered from 825 teachers in 15 basic education schools in China. Respondents were assured of confidentiality, and souvenirs were provided as tokens of appreciation.

Demographic details and gratitude data were collected at Time 1, prosocial motivation and intrinsic motivation at Time 2, and career crafting at the final time. Among respondents, 217 were male, 608 were female, with age distribution as follows: 8.6% were 25 or younger, 34.7% were 25–35, 21.7% were 35–45, and 35.0% were older than 45. Educational level, 2.2% had a college degree or below, 80% were undergraduates, 17% were postgraduates, and 0.8% held doctorates. Regarding tenure, 4.8% had worked for their current school for 3 years or less, 15.9% for 3–7 years, and 49.9% for more than 7 years. Lastly, 10.3% had annual salaries under 50 thousand CNY, 29.1% between 50,000 and 100,000 CNY, 50.2% between 100,000 and 200,000 CNY, and 10.4% more than 200 thousand CNY.

### 3.2. Measures

Following Brislin’s [56] back-translation method, we translated all English scales into Chinese. As shown in Table 1, the composite reliability (CR), Cronbach’s α, and average variance extracted (AVE) were used to assess the internal validity, and the square root of average variance extracted (DV) to examine the discriminant validity [57].

#### 3.2.1. Gratitude

Gratitude was measured through a 6-item scale of McCullough et al. [32] using a 5-point Likert-type scale from 1 (completely disagree) to 5 (completely agree). A sample item was “I am grateful to a wide variety of people”. Cronbach’s α was 0.94, CR was 0.94, AVE was 0.72, and DV was 0.85.

#### 3.2.2. Career Crafting

We measured career crafting using Tims and Akkermans’ [2] 8-item scale rated by a 6-point Likert-type scale from 1 (never) to 6 (always). A sample item was “I assess for myself what I really value in my career”. Cronbach’s α was 0.95, CR was 0.97, AVE was 0.81, and DV was 0.90.

#### 3.2.3. Intrinsic and Prosocial Motivation

Referring to Grant [20], both prosocial and intrinsic motivations were measured by a 7-point Likert-type scale with 4 items, ranging from 1 (completely disagree) to 7 (completely agree), and the introductory question (why are you motivated to do your work?) was also used. The sample item of prosocial motivation was “Because it is important to me to do good for others through my work”, Cronbach’s α was 0.95, CR was 0.95, AVE was 0.83, and DV was 0.91. The sample item of intrinsic motivation was “Because it’s fun.”, Cronbach’s α was 0.96, CR was 0.96, AVE was 0.87, and DV was 0.93.

#### 3.2.4. Control Variables

Drawing from prior research on prosocial motivation and career crafting, we controlled for key demographics (i.e., gender, age, education, tenure, and salary) in alignment with established studies [2,9,20]. For instance, Doerwald et al. [58] highlight the influence of age, tenure, gender, and education on individual motivation and career outcomes. Given gender differences in career progression [8], it is a vital factor in career crafting research. Age may negatively impact career crafting [7], and education level and salary are significant factors linked to career crafting [10].

## 4. Results

### 4.1. Preliminary Analysis

We conducted confirmatory factor analysis (CFA) to test our model fit among the main variables before the hypothesis test. The results (χ^2^/*df* = 2.998; CFI = 0.965; TLI = 0.959; RMSEA = 0.049; SRMR = 0.033) revealed that our theoretical model exhibits a superior fit to the data compared to alternative models (as shown in Table 2), thereby confirming the discriminant validity among the main variables. Table 1 presents the descriptive statistics and correlations. We also assessed multicollinearity by testing the variance inflation factor (VIF) and the figure of tolerance. As shown in Table 3, there was no important issue for our study as the variance inflation factor (VIF) illustrated did not exceed 10, and the tolerance values were all above 0.2, which is accepted as the threshold for regression models [59].

### 4.2. Hypothesis Testing

Table 4 illustrates the results of hierarchical regression analysis using SPSS 26. Gratitude could positively affect prosocial motivation (*b* = 0.55, *p* < 0.001), supporting Hypothesis 1. Subsequently, our findings indicated that prosocial motivation exerts a significant impact on career crafting (*b* = 0.31, *p* < 0.001), and concurrently, the impact of gratitude on career crafting remains significant (*b* = 0.30, *p* < 0.001). This illustrated that gratitude could promote career crafting through the lens of prosocial motivation, supporting Hypotheses 2 and 3. Furthermore, the impact of intrinsic motivation on career crafting was found to be statistically significant (*b* = 0.26, *p* < 0.001), and career crafting was also positively influenced by the interplay of prosocial motivation and intrinsic motivation (*b* = 0.08, *p* < 0.001). The interaction effect on career crafting resulted in an improvement in the model’s coefficient of determination (R^2^) by 8 percent, reaching statistical significance (*p* < 0.01). This indicated that intrinsic motivation had a moderating effect on the relationship between prosocial motivation and career crafting.

**Figure 2:**
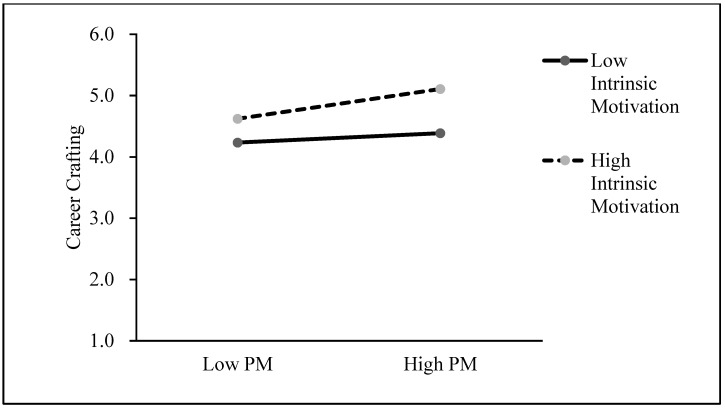
The interaction effect of prosocial motivation and intrinsic motivation on career crafting. *Note.* PM = Prosocial motivation.

Then, we adopted PROCESS [60] to conduct a bootstrap analysis (set as 5000) to further confirm the moderating effect and analyze the different relationships between prosocial motivation and career crafting under different levels of intrinsic motivation (the confidence interval was set as 95%). Table 5 reveals that irrespective of the level of intrinsic motivation, the association between prosocial motivation and career crafting consistently demonstrated a positive and significant relationship. Moreover, with the increase in intrinsic motivation, the effect of prosocial motivation and career crafting increased from 0.08 (LLCI = 0.01, ULCI = 0.16) to 0.260 (LLCI = 0.16, ULCI = 0.36). Furthermore, our simple slope analysis (as presented in Figure 2) revealed that the positive relationship between prosocial motivation and career crafting was enhanced as intrinsic motivation increased. Hence, Hypothesis 4 was supported.

The integrated model was tested by the index of moderated mediation [61] using PROCESS. As shown in Table 6, we grouped intrinsic motivation into low (MEAN − SD) and high (MEAN + 1SD) levels to observe specific mediated effects. When individuals had a low level of intrinsic motivation, the mediating effect of prosocial motivation was not significant (LLCI = −0.01, ULCI = 0.11), and the 95% CI contained 0. Only when employees had a high level of intrinsic motivation could gratitude have a significant effect on career crafting through prosocial motivation. The 95% CI of the mediating effect was (LLCI = 0.05, ULCI = 0.22), excluding 0, and the coefficient was 0.14. The 95% CIs of the difference between the two paths (*b* = 0.01, CI [0.02, 0.17]) and the moderated mediating effect index (*b* = 0.04, CI [0.01, 0.07]) did not include 0. Therefore, a moderated mediated effect existed, and H5 was supported. These findings suggest that individuals with elevated levels of gratitude tend to exhibit heightened prosocial motivation, consequently leading to increased levels of career crafting. Notably, this process is moderated by individual intrinsic motivation.

## 5. Discussion

Career crafting provides individuals with a novel method to adapt to evolving career contexts [2,4]. Despite this, limited research has explored personality-related factors that consistently forecast career proactivity [9]. Moreover, given that careers are intertwined with social networks, career crafting necessitates substantial social engagement. Our study emphasizes the role of gratitude in this context, highlighting its connection to social interactions and its facilitation of interpersonal relationship appreciation, thereby fostering career crafting.

In line with previous research, our study found that gratitude could positively affect prosocial motivation [13,38], and prosocial motivation could be a powerful factor that sustains proactivity (e.g., career crafting) [42]. Therefore, based on the proactive motivation model, this study argues that gratitude can serve as a distal antecedent to motivate proactive behaviors (i.e., career crafting) by facilitating the emergence of prosocial motivation as a “reason to proactivity”.

In addition, the results show that internal motivation serves as our moderating factor, positively influencing this process. This is consistent with the previous view that intrinsic motivation can foster the positive emotions generated by prosocial motivation [52], thus prompting proactive behavior [53]. Intrinsic motivation would amplify the relationship between prosocial motivation and career crafting. Consequently, the findings align with the model of proactive motivation and support our hypothesized moderated mediation model.

### 5.1. Theoretical Implications

First, we identify gratitude as a predictor of career crafting, enriching the existing career crafting literature by linking it to personality traits. Few studies have explored the distal influencing factors of career crafting [62]. However, we cannot ignore this underlying mechanism. Moreover, our research substantiates the presence of the career crafting variable within the model of proactive motivation in the career domain, aligning with previous studies [18,24]. Since gratitude demonstrates a positive trait of expressing appreciation externally and making it easier to establish social connections, we explored how this prosocial trait translates into proactive reflection and construction in one’s career. Our research is consistent with the finding of Xu et al. [19] that proactive personality, as a distal influencing factor of prosocial motivation, affects proactive behavior. This supports the model of proactive motivation by highlighting the significance of distal traits in fostering proactive behaviors.

Second, we delve into the underlying mechanism of prosocial motivations, responding to Parker et al.‘s [14] suggestion for the restructuring of relational work and the provision of structural support. Past research has mostly focused on individuals’ pursuit of self-goals, interests, and values in their careers [1,2,7,9] but has ignored that individuals’ prosocial orientation may also bring benefits to career crafting. Although prosocial motivation is just a motivational state, it reflects an individual’s desire to connect with others and provide help and support. Especially during this process, individuals will be motivated to proactively obtain contact and support for proactive career solutions [43]. Parker et al. [24] noted that even if individuals have the ability and energy to be proactive, they still need a compelling reason to do so. Among the three motivational states, “reason to” motivation is often regarded as the most crucial but has been largely overlooked in research [63,64]. Our study confirms that individuals’ prosocial motivations inspire them to take proactive actions in their careers. This reflects the “reason to” motivation within the proactive motivation model, demonstrating how prosocial goals can drive proactive career behaviors.

Finally, our study reveals the moderator of intrinsic motivation, highlighting that the interaction between intrinsic and prosocial motivations can augment an individual’s career proactivity. Grant [20] demonstrated that the interaction of these two motivations promotes productivity and performance. We draw on this logic and prove that the interaction of these two motivations may affect individual behavior and thereby affect outcomes. We verified that prosocial motivation could help to craft one’s own career, but at the same time, motivation directed to self (i.e., intrinsic motivation) will interact with it to strengthen proactive behavior.

### 5.2. Practical Implications

Individuals undergoing career crafting can benefit in several ways, particularly by cultivating a sense of gratitude and igniting internal motivation. Cultivating gratitude is crucial for enhancing personal well-being and social interactions, fostering prosocial motivation, and actively engaging in social relationships. This appreciation of support and contributions from others provides valuable social support for reshaping one’s career. Simultaneously, clarifying career vision, values, and personal goals ignites internal motivation, aiding in maintaining goal orientation and adaptability during the challenges of career crafting.

For organizations, promoting gratitude and prosocial motives is essential. This can be achieved through training, incentives for positive social interactions, and fostering a culture of appreciation. Creating a supportive work environment that encourages social interactions, networks, and meaningful relationships further provides valuable support for employees engaged in career crafting. Additionally, prioritizing the nurturing of employees’ internal motivation through development opportunities and incentives empowers them to achieve their personal career goals.

Career counselors can leverage gratitude as a catalyst for career crafting by fostering awareness, tailoring interventions to motivational profiles, and promoting prosocial values. This approach enhances clients’ proactive engagement with their evolving career paths and contributes to their ability to navigate dynamic career landscapes. Integrating gratitude into career counseling practices is pivotal for the holistic development of individuals’ career-crafting capabilities.

### 5.3. Limitations and Future Research Directions

In addition to the above contributions, some limitations need to be discussed. First, although we have studied the antecedents that affect careers from the perspective of social interaction, this study only focuses on the interaction between individual motivations and does not take into account contextual variables in organizations or variables of individual interactions. Future research could explore the impact of situational variables such as job characteristics, interactions with others such as leader–member exchange, and feedback from service clients on individuals’ careers in this process. Second, individuals are complex and may possess many characteristics. Future studies may consider the impact of other individual traits, such as extraversion, on career crafting and further explore the important role of subtypes of individual traits in individual careers through other methods, such as potential profile analysis. Third, we use the time-lagged data for analysis in our paper to avoid common method bias, but the causal effect could not be tested. At the same time, since a career is a relatively long-term process, we suggest that follow-up studies should be conducted to test causality through longitudinal studies or experiments.

## 6. Conclusions

Drawing on a model of proactive motivation, our study highlights the mechanism by which gratitude, as a distal antecedent, contributes to proactive behavior (i.e., career crafting) by influencing prosocial motivation, which is the “reason to proactivity”. We found that intrinsic motivation plays a key moderating role in this process (i.e., this mediating role can only be established when intrinsic motivation is at a high level). Therefore, our study can not only guide individuals in their career development, which can benefit them by fostering gratitude and stimulating intrinsic motivation, but also provide organizations with new ideas for training. At the same time, career counselors can also view gratitude as a catalyst for career acceleration by increasing gratitude awareness and adapting interventions to the individual’s internal motivation profile.

## Figures and Tables

**Table 1:** Descriptive statistics, correlations, reliability, and discriminant validity.

Variables	Mean	SD	α	CR	AVE	1	2	3	4
1 Gratitude	4.19	0.60	0.94	0.94	0.72	**(0.85)**			
2 Prosocial motivation	5.94	0.93	0.95	0.95	0.83	0.36 **	**(0.91)**		
3 Intrinsic motivation	5.70	1.13	0.96	0.96	0.87	0.30 **	0.72 **	**(0.93)**	
4 Career crafting	4.65	0.84	0.95	0.97	0.81	0.34 **	0.42 **	0.48 **	**(0.90**)

Note. *N* = 825. ** *p* < 0.01. The bold values in the parentheses on the diagonal of the matrix are the square root of AVE. α = Cronbach alpha.

**Table 2:** Model fit indicator results.

	χ^2^	*df*	χ^2^*/df*	CFI	TLI	SRMR	RMSEA
4-factor model	602.541	201	2.998 ***	0.965	0.959	0.033	0.049
3-factor model	1662.969	204	8.152 ***	0.871	0.854	0.055	0.093
2-factor model	3639.526	206	17.668 ***	0.697	0.66	0.154	0.142
Single factor model	6421.578	209	30.725 ***	0.451	0.393	0.189	0.19

Note. *N* = 825. *** *p* < 0.001. 4-factor model: Gratitude, Prosocial motivation, Intrinsic motivation, and Career Crafting; 3-factor model: Gratitude, Prosocial motivation + Intrinsic motivation, Career Crafting; 2-factor model: Gratitude + Prosocial motivation + Intrinsic motivation, Career Crafting; Single-factor model: Gratitude + Prosocial motivation + Intrinsic motivation + Career Crafting.

**Table 3:** VIF scores and tolerance levels.

Variable	Tolerance Level	VIF Score
Gratitude	0.827	1.209
Prosocial motivation	0.381	2.626
Intrinsic motivation	0.468	2.138

Note. *N* = 825.

**Table 4:** Results of regression analysis.

Variables	Prosocial Motivation		Career Crafting				
	M1	M2	M3	M4	M5	M6	M7
Control variables							
Gender	0.14 (0.07)	0.13 (0.06)	−0.21 ** (−0.11)	−0.22 ** (−0.12)	−0.26 *** (−0.14)	−0.24 *** (−0.12)	−0.20 *** (−0.11)
Age	0.05 (0.05)	0.05 (0.05)	0.04 (0.05)	0.04 (0.05)	0.03 (0.04)	0.00 (0.01)	0.02 (0.02)
Education level	0.10 (0.05)	0.06 (0.03)	0.17 * (0.09)	0.14 * (0.07)	0.12 (0.06)	0.10 (0.05)	0.09 (0.05)
Tenure	−0.01 (−0.01)	0.02 (0.02)	−0.05 (−0.05)	−0.02 (−0.02)	−0.02 (−0.03)	−0.01 (−0.01)	−0.03 (−0.03)
Salary	0.09 (0.08)	0.00 (0.00)	0.09 * (0.09)	0.02 (0.02)	0.02 (0.02)	0.03 (0.02)	0.02 (0.02)
Predictors							
Gratitude		0.55 *** (0.36)		0.47 *** (0.34)	0.30 *** (0.22)	0.28 *** (0.20)	0.25 *** (0.18)
Prosocial motivation					0.31 *** (0.34)	0.09 * (0.10)	0.17 *** (0.19)
Intrinsic motivation						0.26 *** (0.35)	0.25 *** (0.33)
PM × IM							0.08 *** (0.16)
R2	0.02	0.14	0.030	0.13	0.24	0.29	0.32
ΔR2	0.02	0.12	0.030	0.11	0.10	0.06	0.02
F-value	2.49 *	115.22 ***	4.17 **	103.17 ***	108.332 **	67.72 ***	24.47 ***

Note. *N* = 825. All regression coefficients are unstandardized. * *p* < 0.05, ** *p* < 0.01, *** *p* < 0.001. PM × IM = Prosocial motivation × Intrinsic motivation. The standardized coefficients are in parentheses, and the significance of the coefficients inside and outside the parentheses is the same. (1) Gender is coded as 1 = Male and 2 = Female. (2) Age (in years) is coded as 1 = ≤25, 2 = 26–35, 3 = 36–45, 4 = >45. (3) Education level is coded as 1 = College degree or below, 2 = Bachelor’s degree, 3 = Postgraduate degree, 4 = Doctoral degree. (4) Tenure (in years) is coded as 1 = ≤3, 2 = 3–7, 3 = >7. (5) Salary (RMB) is coded as 1 = <50,000, 2 = 50,000–100,000, 3 = 100,001–20,000, 4 = >20,000. Source(s): Authors work.

**Table 5:** Relationship between prosocial motivation and career crafting under different levels of intrinsic motivation.

Intrinsic Motivation	Effect	*SE*	*t*-Value	*p*-Value	LLCI	ULCI
Low level (MEAN − 1SD)	0.08	0.04	2.10	0.04	0.01	0.16
Medium level (MEAN)	0.17	0.04	4.02	0.00	0.09	0.25
High level (MEAN + 1SD)	0.26	0.05	4.96	0.00	0.16	0.36

Note. *N* = 825. Bootstrap = 10,000, 95% confidence interval.

**Table 6:** The moderating effect of intrinsic motivation on the mediating effect of prosocial motivation.

Intrinsic Motivation	Effect	*SE*	BootLLCI	BootULCI
	0.25	0.04	0.16	0.34
	Effect	BootSE	BootLLCI	BootULCI
Low level (MEAN − 1SD)	0.05	0.03	−0.01	0.11
High level (MEAN + 1SD)	0.14	0.04	0.07	0.22
Difference (low and high)	0.10	0.04	0.02	0.17
Moderated mediation effect index	0.04	0.02	0.01	0.07

Note. *N* = 825. Bootstrap = 10,000, 95% confidence interval.

## Data Availability

The data presented in this study are available on request from the corresponding author due to participant privacy, ethical considerations, and confidentiality obligations. Access to the data is restricted to ensure the protection of personal information and to comply with ethical guidelines and confidentiality agreements. Interested researchers may contact the corresponding author to request access, providing a detailed explanation of the intended use and demonstrating adherence to the ethical and confidentiality standards outlined in this study.

## Appendix A. The Engagement Scales

**Gratitude** [32]

1. I have so much in life to be thankful for.

2. If I had to list everything that I felt grateful for, it would be a very long list.

3. When I look at the world, I do not see much to be grateful for.

4. I am grateful to a wide variety of people.

5. As I get older, I find myself more able to appreciate the people, events, and situations that have been part of my life history.

6. Long amounts of time can go by before I feel grateful for something or someone.

Items 3 and 6 are reverse-scored.

**Career crafting** [2]

1. I spend time reflecting on my passions in my work and career.

2. I deliberately think about what I would like to achieve in my career.

3. I assess for myself what I really value in my career.

4. I explore the possibilities available to me to continue developing myself.

5. I make sure that significant persons in my work are up to date about my performance and results.

6. I deliberately show others what I am good at.

7. I strengthen my career goals and make sure that they remain up to date.

8. If I need to make a strong impression on others to attain my own goals, I make sure I clearly show them what I am capable of.

**Intrinsic and Prosocial motivation** [20]

Why are you motivated to do your work?

**Prosocial motivation:**

1. Because I care about benefiting others through my work.

2. Because l want to help others through my work.

3. Because I want to have a positive impact on others.

4. Because it is important to me to do good for others through my work.

**Intrinsic motivation:**

1. Because I enjoy the work itself.

2. Because it is fun.

3. Because I find the work engaging.

4. Because I enjoy it.

**Table A1:** Reliability analysis.

Variables	Items	STD.	S.E.	Z	*p*-Value	SMC
GRA	G1	0.869	0.014	61.613	0.000	0.755
	G2	0.857	0.017	51.835	0.000	0.734
	G3	0.902	0.012	77.035	0.000	0.814
	G4	0.838	0.018	45.393	0.000	0.702
	G5	0.809	0.022	36.190	0.000	0.654
	G6	0.798	0.020	40.564	0.000	0.637
PROM	PROM1	0.815	0.032	25.777	0.000	0.664
	PROM2	0.957	0.011	87.320	0.000	0.916
	PROM3	0.953	0.010	98.466	0.000	0.90
	PROM4	0.914	0.015	62.923	0.000	0.835
INTM	INTM1	0.923	0.009	100.423	0.000	0.852
	INTM2	0.930	0.010	96.641	0.000	0.865
	INTM3	0.929	0.013	73.344	0.000	0.863
	INTM4	0.952	0.008	113.947	0.000	0.906
CC	CC1	0.905	0.012	76.666	0.000	0.819
	CC2	0.930	0.012	76.182	0.000	0.865
	CC3	0.926	0.010	92.713	0.000	0.857
	CC4	0.915	0.009	97.485	0.000	0.837
	CC5	0.883	0.013	65.963	0.000	0.780
	CC6	0.885	0.015	59.159	0.000	0.783
	CC7	0.848	0.020	42.560	0.000	0.719
	CC8	0.899	0.012	76.324	0.000	0.808
